# Collaborative Depression Care Among Latino Patients in Diabetes Disease Management, Los Angeles, 2011–2013

**DOI:** 10.5888/pcd11.140081

**Published:** 2014-08-28

**Authors:** Brian Wu, Haomiao Jin, Irene Vidyanti, Pey-Jiuan Lee, Kathleen Ell, Shinyi Wu

**Affiliations:** Author Affiliations: Brian Wu, Haomiao Jin, Irene Vidyanti, Pey-Jiuan Lee, Kathleen Ell, University of Southern California, Los Angeles, California; Shinyi Wu, RAND Corporation, Santa Monica, California.

## Abstract

**Introduction:**

The prevalence of comorbid diabetes and depression is high, especially in low-income Hispanic or Latino patients. The complex mix of factors in safety-net care systems impedes the adoption of evidence-based collaborative depression care and results in persistent disparities in depression outcomes. The Diabetes–Depression Care-Management Adoption Trial examined whether the collaborative depression care model is an effective approach in safety-net clinics to improve clinical care outcomes of depression and diabetes.

**Methods:**

A sample of 964 patients with diabetes from 5 safety-net clinics were enrolled in a quasi-experimental study that included 2 arms: usual care, in which primary medical providers and staff translated and adopted evidence-based depression care; and supportive care, in which providers of a disease management program delivered protocol-driven depression care. Because the study design established individual treatment centers as separate arms, we calculated propensity scores that interpreted the probability of treatment assignment conditional on observed baseline characteristics. Primary outcomes were 5 depression care outcomes and 7 diabetes care measures. Regression models with propensity score covariate adjustment were applied to analyze 6-month outcomes.

**Results:**

Compared with usual care, supportive care significantly decreased Patient Health Questionnaire-9 scores, reduced the number of patients with moderate or severe depression, improved depression remission, increased satisfaction in care for patients with emotional problems, and significantly reduced functional impairment.

**Conclusion:**

Implementing collaborative depression care in a diabetes disease management program is a scalable approach to improve depression outcomes and patient care satisfaction among patients with diabetes in a safety-net care system.

## MEDSCAPE CME

Medscape, LLC is pleased to provide online continuing medical education (CME) for this journal article, allowing clinicians the opportunity to earn CME credit.

This activity has been planned and implemented in accordance with the Essential Areas and policies of the Accreditation Council for Continuing Medical Education through the joint sponsorship of Medscape, LLC and Preventing Chronic Disease. Medscape, LLC is accredited by the ACCME to provide continuing medical education for physicians.

Medscape, LLC designates this Journal-based CME activity for a maximum of 1 **AMA PRA Category 1 Credit(s)™**. Physicians should claim only the credit commensurate with the extent of their participation in the activity.

All other clinicians completing this activity will be issued a certificate of participation. To participate in this journal CME activity: (1) review the learning objectives and author disclosures; (2) study the education content; (3) take the post-test with a 75% minimum passing score and complete the evaluation at www.medscape.org/journal/pcd (4) view/print certificate.

**Release date: August 28, 2014; Expiration date: August 28, 2015**

### Learning Objectives

Upon completion of this activity, participants will be able to:

- Distinguish psychosocial variables improved with a supportive care program
- Compare the effects of a supportive care program on outcomes related to diabetes and depression
- Assess the effects of a supportive care program on outcomes related to medical disease

**EDITOR**

Camille Martin, Technical Writer/Editor, *Preventing Chronic Disease*. Disclosure: Camille Martin has disclosed no relevant financial relationships.

**CME AUTHOR**

Charles P. Vega, MD, Clinical Professor of Family Medicine, University of California, Irvine. Disclosure: Charles P. Vega, MD, has disclosed the following relevant financial relationships: Served as an advisor or consultant for: McNeil Pharmaceuticals.

**AUTHORS AND CREDENTIALS**

Disclosures: Brian Wu, BS; Haomiao Jin, MS; Irene Vidyanti, MEng; Pey-Jiuan Lee, MS; Kathleen Ell, DSW; Shinyi Wu, PhD have disclosed no relevant financial relationships.

Affiliations: Brian Wu, Haomiao Jin, Irene Vidyanti, Pey-Jiuan Lee, Kathleen Ell, University of Southern California, Los Angeles, California; Shinyi Wu, RAND Corporation, Santa Monica, California.

## Introduction

Diabetes is a chronic, lifelong illness that increases the risk of illness and death (1). Diabetes doubles the risk of comorbid depression (2,3). The high prevalence of depression with concurrent diabetes increases patient disability and need for social support and negatively affects treatment efficacy, medication adherence, self-care management, patient–physician communication, and quality of life (4–6). Furthermore, Hispanics and Latinos have a higher prevalence of diabetes than non-Hispanic or non-Latino whites (7), and patients with comorbid depression and diabetes are at greater risk of functional disability, poor health service use, and death (8,9).

Primary care depression treatment is effective among low-income, racial and ethnic minority populations (10–13). When collaborative care is adopted both by patients and providers, the treatment of depression becomes effective and cost-efficient (14). However, the complex mix of patient, provider, and health system factors in safety-net care systems impedes the adoption of evidence-based collaborative depression care and results in persistent disparities in depression outcomes.

The Diabetes–Depression Care-Management Adoption Trial (DCAT) examined a safety-net disease management program for depression prevention, screening, surveillance, and intervention (15). There are many single-disease management programs, including many for diabetes. These programs provide the infrastructure of care teams and patient registries for implementing collaborative depression care. Supportive care programs that expand a single-disease focus to concurrently address the common comorbid condition of depression have the potential to better meet patient needs and improve clinical outcomes (14,16,17). 

We examined the ability of a supportive care approach to fill gaps in the implementation of depression care and facilitate optimal adaptive depression care management in safety-net primary care settings. We expected that DCAT would find a supportive care program improves 6-month clinical outcomes of both diabetes and depression.

## Methods

### Overall design, intervention, and hypothesis

The DCAT team conducted a quasi-experimental trial that examined the effects of implementing depression monitoring in a diabetes disease management program for low-income urban populations in the Los Angeles County Department of Health Services (DHS) Ambulatory Care Network, the second-largest safety-net care system in the United States. Before DCAT, DHS had a diabetes disease management program with nurse-driven and physician-supervised care management for high-risk or high-service-use patients. DHS applies evidence-based diabetes care management components and uses structured tools (case management, patient education and self-management support, care coordination, depression screening and physician notification, an electronic disease registry, and integrated clinical decision support systems) to deliver more than 80% of the care by nurses under protocol and was the model for the supportive care group. These tools support clinical assessment and decisions in a limited care-management period of 6 months. The integration of team staff, including physicians, nurse practitioners, nurses, and social workers, provided an intensive care model with strict guidelines for follow-up and monitoring of diabetes symptoms and comorbid risks, such as depression. Participants received weekly telephone calls from care team members and were seen by nurses and social workers who provided comprehensive team-based care to improve disease management and quality of care.

During implementation of DCAT, from October 2011 to May 2013, diabetes disease management was supplemented with periodic screening and monitoring of depression symptoms with the Patient Health Questionnaire 9-item scale (PHQ-9), a standard tool in each clinic’s disease registry, and the DHS depression care protocol and treatment guideline. The program also designated a social worker to provide problem-solving therapy, an evidence-based treatment of depression. All care providers were offered training in problem-solving therapy via a 1-day workshop; they were also trained in the collaborative depression care model and adaptive treatment approach via 1 of 3 webinars.

This study involved 5 DHS primary care clinics, selected by DHS leaders on the basis of criteria that reflected geographic and diabetes care model diversity. The usual care group included 2 community clinics that represented standard clinical practice, in which primary medical providers and their staff translated and adopted evidence-based depression care. The supportive care study group included 2 care teams from the DHS diabetes disease management program. These teams practiced in 2 community clinics and 1 hospital-based outpatient clinic. We hypothesized that at 6 months after enrollment in the study, patients who received the diabetes supportive care would have improved depression and diabetes outcomes compared with patients receiving usual care.

### Population characteristics and eligibility

Patients were recruited from 5 DHS primary care clinics. The patients were predominantly low-income, low-literacy, middle-aged, Spanish-speaking Hispanic or Latino women who had been diagnosed with diabetes for more than 5 years. Approximately one-third of participants were depressed, and approximately one-third of the patients were men.

Patients were eligible for the study if they were aged 18 years or older with type 2 diabetes, had a working telephone number, spoke English or Spanish, and read and understood the consent form. Patients were ineligible for the trial if they presented with baseline acute suicidal ideation (as measured by PHQ-9, item 9), cognitive impairment (Short Portable Mental Status Questionnaire scores less than 5) (18), alcohol abuse (2 or more CAGE items from a quantity–frequency index, and patient perceptions of substance use) (19), or if they had recently used lithium or antipsychotic medication. Patients were not required to have depression to be eligible for the study because DCAT addressed the elevated risk of depression among patients with diabetes by testing a care approach that incorporates depression screening, symptom monitoring, and treatment follow-up for diabetes patients.

### Recruitment

Approval was obtained from the University of Southern California and the Los Angeles Biomedical Research Institute human subjects review boards. The enrollment period was from April 2011 to May 2012 in the 5 study clinics. Patients with type 2 diabetes were identified for recruitment from database and clinic records. Patients provided verbal consent during study eligibility screening to bilingual research assistants. Of the 1,704 patients screened, 1,066 (63%) were women and 638 (37%) were men. Men had a significantly lower enrollment rate than women (83% vs 88%, respectively; *P* = .003), which was associated with poor alcohol use scores (5% vs 1%, respectively). A total of 964 diabetes patients (86% of patients screened) provided written informed consent and completed a structured baseline interview that included both PHQ-9 (scores range from 0–27, where higher scores indicate worse depression) and Hopkins Symptom Checklist-20 depression symptom assessments (individual items scored 0–4, where higher scores indicate worse depression) (Figure). After excluding 87 participants with missing data, the baseline sample had 877 patients: 416 patients in the usual care group and 461 patients in the supportive care group. Patient and family educational materials concerning depression, including a comic book *fotonovela* (20) designed for patients and family members with low health literacy, were provided to all study patients in Spanish or English by bilingual study recruiters. The study clinic physicians were notified of the baseline depression screening results for patients whose PHQ-9 scores were 10 or higher or who exhibited suicidal ideation (score greater than 1 for item 9 of PHQ-9). Participants received no monetary incentive for the study enrollment and baseline assessment. However, they did receive a $10 gift card for each follow-up assessment they completed. The study clinics participated in the study pro bono.

**Figure:**
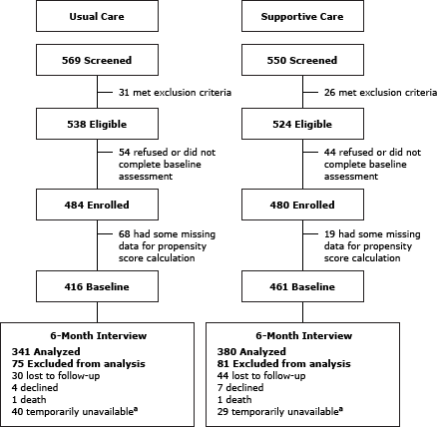
Consolidated Standards of Reporting Trial (CONSORT) diagram of sample of study participants drawn from type 2 diabetes patients identified in database and clinic records at safety-net clinics where they sought treatment, Los Angeles County, California, 2011–2013. Propensity scores were used to determine the probability of treatment assignment conditional on observed baseline characteristics. Some patients were excluded because they were temporarily unavailable (eg, they were out of the state or country, it was not a good time to talk, their telephone was disconnected).

### Outcome measures

All subjects received comprehensive assessments at baseline and at 6, 12, and 18 months by independent English–Spanish bilingual interviewers. Primary outcomes included 5 depression outcomes and 7 diabetes care measures (including satisfaction with care and disability reduction) (Table 1).

**Table 1:** Primary Outcome Measures, Diabetes–Depression Care-Management Adoption Trial, Los Angeles, 2011–2013

Outcome	Description
**Depression**	
PHQ-9	A continuous variable that assesses severity of depression.
PHQ-9 ≥10	A dichotomous variable that assesses severity of depression. PHQ-9 ≥10 indicates major depression. Higher scores indicate worse depression.
Depression remission	A dichotomous variable that assesses effectiveness of treating patients with major depression. Depression remission is defined as baseline PHQ-9 ≥10 and 6-month PHQ-9 ≤8 with a reduction ≥50%.
Satisfaction in care with emotional problems	Five-level score that assesses mental care satisfaction. Treated as continuous variable.
Satisfaction in care with emotional problems for patients with baseline PHQ-9 ≥10	Five-level score that assesses mental care satisfaction of patients with major depression. Treated as continuous variable.
**Diabetes**	
A1c value	A continuous variable that assesses severity of diabetes. A1c value indicates the average plasma glucose concentration over prolonged periods.
A1c tested	A dichotomous variable that assesses the diabetes care process.
Total cholesterol	A continuous variable that evaluates cholesterol levels and severity of diabetes.
Diabetes self-care	Number of days per week of diabetes self-care. Treated as a continuous variable.
Exercise	Number of days of exercise during the previous week.
Sheehan Disability Scale	A self-report tool that assesses functional impairment in work or school, social, and family life.
Satisfaction in diabetes care	Five-level score that assesses diabetes care satisfaction. Treated as continuous variable.

Abbreviations: PHQ-9, Patient Health Questionnaire-9; A1c, glycated hemoglobin.

### Sample size calculation

The target sample size was based on power analysis for 2 primary outcomes: reduction of prevalence of major depression (PHQ-9 score ≥10) and depression remission (PHQ-9 score ≤8 with a reduction ≥50% for patients with major depressive disorder at baseline). Power analyses were conducted using nQuery (Statistical Solutions) to estimate effect sizes of the treatment with preintervention and postintervention comparisons and longitudinal statistical approaches for repeated measures comparing the trend of depression-related outcomes in the DCAT study. The calculations assumed an α level of .05 and a power of .80. With the assumption that attrition rates would be less than 20% for patients at each 6-month follow-up assessment — up to 18 months for preintervention and postintervention comparisons — a sample size of 51 patients with depression in each study group would allow the detection of a small effect size of less than .01. A previous trial in 2008 of the Multifaceted Depression and Diabetes Program established that 25% to 30% of diabetes patients also experience depression (21); for DCAT, we added a 25% cushion for differences in baseline characteristics because of the quasi-experimental trial design. Therefore, DCAT required a sample size of approximately 500 type 2 diabetes patients in each study group.

### Statistical methods

Initial statistical tests were performed to assess differences in differences (DID). However, because the study design defined individual treatment centers as separate arms, we aimed to improve statistical testing by calculating propensity scores to interpret the probability of treatment assignment conditional on observed baseline characteristics. Both tests were performed with assumptions of *P* ≤ .05. A multinomial logistic regression model was used to estimate the propensity score; the model used study group as the dependent variable and all 27 measured baseline characteristics as the independent variables. The baseline characteristics were 1) age, 2) sex, 3) preferred language, 4) body mass index, 5) education level, 6) employment status, 7) economic status, 8) total stressor number, 9) sum of the stress level, 10) predicted future health cost, 11) age at onset of diabetes, 12) insulin use, 13) Medical Outcomes Study Short Form-12 Health Survey (SF-12) physical score (scored 0–100, where higher scores indicate a higher level of physical health), 14) SF-12 mental score (scored 0–100, where higher scores indicate a higher level of mental health), 15) number of diabetes complications, 16) Whitty-9 diabetes symptoms scale score (scored 1–5, where higher scores indicate more severe diabetes), 17) diabetes emotional burden, 18) diabetes regimen distress, 19) mean Toolbert diabetes self-care score (scored 0–7, where higher scores indicate better diabetes self-care), 20) PHQ-9 score (scored 0–27, where higher scores indicate worse depression), 21) Brief Symptom Inventory total score (scored 0–24, where higher scores indicate worse anxiety) , 22) mean Sheehan Disability Scale score (scored 0–10, where higher scores indicate more significant functional impairment), 23) dysthymia, 24) previous diagnosis of major depressive disorder, 25) chronic pain, 26) overall patient satisfaction, and 27) glycated hemoglobin (A1c) value. We subsequently checked the distribution of the estimated propensity scores because between-group comparisons would be suspect if there had been substantial separation between treatment arms; the propensity score method is a more conservative and parsimonious statistical modeling method for nonrandomized study samples (22).

Comparative treatment effects were estimated using linear or logistic regression models featuring outcomes at 6 months as the dependent variable; the independent variables were study group, care team, outcome variable at baseline, estimated propensity scores, insulin use, A1c, age, sex, and preferred language. Regression that includes estimated propensity scores as covariates is an effective tool to adjust sample biases in observational or quasi-experimental studies (23). The coefficients of study groups predicted comparative treatment effects. Three care team variables were used to adjust for differences among providers. Analyses were performed by using SAS version 9.1 (SAS Institute, Inc).

## Results

### Population characteristics

The DCAT study enrolled 964 low-income, predominantly Hispanic or Latino patients with diabetes to test and compare the translational models of depression care management. Of these patients, 484 were in the usual care group and 480 in the supportive care group. 

Because DCAT used a quasi-experimental design comparing study groups, we first examined whether major baseline characteristics that could influence the outcome measures were balanced between the study groups. There were no significant differences in baseline PHQ-9 depression or SF-12 mental scores, Sheehan Disability Scale ratings, or body mass index in pairwise comparisons between groups (Table 2). 

**Table 2:** Comparison of Baseline Characteristics of 964 Subjects Enrolled in Diabetes–Depression Care-Management Adoption Trial, Los Angeles, 2011–2013

Characteristics	Usual Care (n = 484)	Supportive Care (n = 480)	*P* Value
**Demographics**			
Female, %	69	59	.002
Age, mean, y	55.0	52.1	<.001
Hispanic or Latino, %	94	83	<.001
Prefers Spanish, %	89	78	<.001
**Diabetes**			
Age at onset of diabetes, mean, y	45.0	41.8	<.001
Uses insulin, %	26	63	<.001
Has diabetes complication, %	71	74	.32
Diabetes self-care, mean	4.00	4.75	<.001
Body mass index, mean, kg/m^2^	32.34	32.55	.66
Sheehan Disability Scale,^a^ mean	2.24	2.13	.55
**Depression and anxiety, mean score**			
Patient Health Questionnaire-9^b^	6.67	6.93	.50
Hopkins Symptom Checklist-20^c^	0.56	0.64	.08
Brief Symptom Inventory^d^	1.35	1.30	.81
**Functional status, Medical Outcomes Study Short Form-12, mean score^e^**			
Physical	43.04	45.81	<.001
Mental	50.05	49.03	.23

a The Sheehan Disability Scale is scored from 0 to 10, where higher scores indicate more significant functional impairment.

b The Patient Health Questionnaire-9 is scored from 0 to 27, where higher scores indicate worse depression.

c Individual items on the Hopkins Symptom Checklist-20 are scored from 0 to 4, where higher scores indicate worse depression.

d The Brief Symptom Inventory is scored from 0 to 24, where higher scores indicate worse anxiety.

e The Medical Outcomes Study Short Form-12 is scored from 0 to 100, where higher scores indicate a higher level of physical health.

### Outcomes

The DID test with the main outcome variable of PHQ-9 change score at 6 months was significant (*P* = .01). When adjusted for the same covariates, DID and propensity score methods had consistent results (*P* = .01 and *P* = .05, respectively). 

Compared with usual care, supportive care significantly decreased PHQ-9 scores (least squares mean [LSM] = 6.34, standard error [SE] = 0.49 vs LSM = 5.08, SE = 0.48, respectively; *P* = .047), reduced the number of patients with moderate or severe depression (a PHQ-9 score ≥10; *P* = .04), and improved depression remission (*P* = .05) (Table 3). Supportive care also significantly improved patient satisfaction with care for emotional problems (*P* = .01). Scores on the Sheehan Disability Scale (*P* = .03) were significantly lower in the supportive care group compared with the usual care group. There were no significant differences between the 2 groups in terms of satisfaction with care, emotional distress among patients with a baseline PHQ-9 score of 10 or more, cholesterol, diabetes self-care, exercise, satisfaction with diabetes care, or A1c levels.

**Table 3:** Regression Analysis of Outcomes^a^, Diabetes–Depression Care-Management Adoption Trial, Los Angeles, 2011–2013

Continuous Outcome	Usual Care, LSM (SE)	Supportive Care, LSM (SE)	*P* Value
PHQ-9 (higher scores indicate worse depression)	6.34 (0.49)	5.08 (0.48)	.047
Satisfaction with emotional care (higher scores indicate greater satisfaction)	3.24 (0.10)	3.64 (0.10)	.01
Satisfaction with emotional care among patients with baseline PHQ-9 score ≥10 (higher scores indicate greater satisfaction)	3.18 (0.22)	3.59 (0.21)	.19
Cholesterol, mg/dL	176.21 (5.24)	166.80 (4.98)	.19
Diabetes self-care (days/week)	4.67 (0.13)	4.70 (0.12)	.93
Exercise (days/week)	4.74 (0.28)	4.90 (0.27)	.64
Sheehan Disability Scale (higher scores indicate greater disability)	3.21 (0.26)	2.60 (0.25)	.03
Satisfaction with diabetes care (higher scores indicate greater satisfaction)	4.00 (0.09)	4.15 (0.09)	.32
A1c value	7.95 (0.17)	7.79 (0.16)	.17

**Dichotomous Outcome**	**Supportive vs Usual Care, OR (95% CI)**		***P* Value**

PHQ-9 score ≥10	0.46 (0.23–0.90)		.04
Depression remission	3.08 (1.01–9.45)		.05
A1c tested	1.80 (0.88–3.68)		.10

Abbreviations: LSM, least squares mean; SE, standard error; PHQ-9, Patient Health Questionnaire-9; A1c, glycated hemoglobin; OR, odds ratio; CI, confidence interval.

a Both linear and logistic regression models were adjusted for study group, care team, outcome variable at baseline, propensity score, insulin use, A1c, age, sex, and preferred language.

## Discussion

Evidence-based collaborative depression care in a diabetes disease management program designed to reduce disparities in combined diabetes and depression care represents an important and valuable tool for future providers that may greatly improve overall care, cost, and effectiveness of health care delivery for underserved patients. Our findings indicated that supportive care through depression monitoring can improve diabetes and depression outcomes in the second-largest US safety-net health system. 

Patients enrolled in the supportive care group had significantly decreased PHQ-9 scores, reduced levels of moderate or severe depression, and improved depression remission. Additionally, patients enrolled in the supportive care group had significantly decreased values on the Sheehan Disability Scale. Although some aspects of care were not significantly different between groups, the ability to target depression as an outcome in a group of patients with diabetes is a potentially life-altering improvement for each patient. Many studies have outlined the risk of worsening diabetes outcomes in patients who also have depression (8,24). These risks are amplified in patients who are unable to receive depression management support in usual care, and these people are often low-income minority patients (7,25). Although between-group changes in A1c levels were not significant, the supportive care group did have lower values. Perhaps an extended study (follow-up beyond 6 months) or a more intensive version of supportive care could promote greater improvement in specific diabetes outcomes. Alternatively, because study patients in the supportive care group had been in the diabetes care management program for 3 months, on average, before the DCAT intervention, diminished improvements in outcomes may have resulted from previous program effects. However, it appears that the care received by the supportive care group resulted in improvements in depression management and may result from the strict guidelines of the diabetes management program in terms of requisite number of visits and follow-up. It may also be that the increased role of nurses and social workers on the care team can provide additional quality and quantity of care, allowing patients to focus on comorbid conditions such as depression.

Applied on a large scale across many different chronic diseases, the integration of care for comorbid diseases could greatly improve outcomes overall and help prevent worsening of chronic diseases. Specifically, decreasing the prevalence of depression in cancer patients significantly improves quality of life and cancer outcomes (10,12). Expanding methods to improve quality of life and clinical outcomes for patients with diabetes, which affects more than 285 million adults worldwide (26), is an additional step to preventing chronic disease progression.

The main limitation of this study is that it was not randomized but rather conducted across 5 DHS clinics as a quasi-experimental trial. However, use of the propensity scores provided an analysis of differences across clinics and suggested that there was no significant difference between sites. Nevertheless, differences across clinics, patients, and providers must be considered for practical application. Another potential limitation may be the focus on a predominantly Hispanic or Latino population. Conversely, an important aspect of the DCAT model was its focus on reducing disparities among low-income minority patients in safety-net primary care settings. The fact that continuous depression symptom assessment, treatment monitoring, and relapse prevention may be difficult in busy safety-net primary care practices may be another limitation; additional methods may be necessary to ensure adoptability, cost-effectiveness, and scalability. However, improvements in clinical satisfaction and outcomes indicate that researchers should study chronic diseases across multiple risks. As such, the design of DCAT diabetes-depression supportive care may be a tool to aid in prevention efforts and care for all patients with chronic diseases. Although some demographic and diabetes variables varied significantly between the study groups, this was expected given that in the quasi-experimental DCAT design, pretreatment differences are more common than those expected from randomized experimental design. However, because participants were recruited from community clinics, results should be applicable to individuals not involved in the study but who also receive community care.

Expanding diabetes disease management to support the incorporation of a collaborative depression care model may be an effective approach to prevent the progression of chronic diseases. Implementing this approach in an underserved population with a high prevalence of diabetes may also have the added benefit of reducing health disparities while improving clinical outcomes and fundamentally influencing primary care. Further research is required to understand the full adaptability of the DCAT supportive care model and its effect on lifetime clinical outcomes.

## Figures and Tables

**Table:** 

**1. The activity supported the learning objectives.**				
**Strongly Disagree**				**Strongly Agree**
1	2	3	4	5
**2. The material was organized clearly for learning to occur.**				
**Strongly Disagree**				**Strongly Agree**
1	2	3	4	5
**3. The content learned from this activity will impact my practice.**				
**Strongly Disagree**				**Strongly Agree**
1	2	3	4	5
**4. The activity was presented objectively and free of commercial bias.**				
**Strongly Disagree**				**Strongly Agree**
1	2	3	4	5

## Post-Test Questions

### Article Title: Collaborative Depression Care Among Latino Patients in Diabetes Disease Management, Los Angeles, 2011–2013

**CME Questions**

1. You are seeing a 54-year-old woman with a history of poorly controlled type 2 diabetes. She complains of insomnia, anxiety, and a depressed mood, and a Patient Health Questionnaire 9-item scale confirms probable depression. You consider referring this patient to a supportive care program similar to the one in the current study. Compared with usual care, which one of the following psychosocial outcomes was ***most*** improved with supportive care in the current study?
  - Anxiety scores
  - Scores on the Patient Health Questionnaire 9-item scale
  - Interpersonal relationships
  - Self-efficacy in managing stress
2. Overall, which one of the following outcomes were improved ***most*** significantly with the supportive care program in the current study?
  - Depression outcomes were improved more than diabetes outcomes
  - Diabetes outcomes were improved more than depression outcomes
  - Both diabetes and depression outcomes were substantially improved
  - Neither diabetes nor depression outcomes were improved
3. Which one of the following outcomes for medical disease was ***most*** improved by the supportive care program in the current study?
  - Disability scale
  - Cholesterol levels
  - Diabetes self-care
  - Hemoglobin A1c levels

**Evaluation**
